# Gender differences in occupational hazard exposures within the same occupation: A nationally representative analysis in South Korea

**DOI:** 10.5271/sjweh.4204

**Published:** 2025-03-01

**Authors:** Garin Lee, Karen Messing, Woojoo Lee, Ji-Hwan Kim, Hayoung Lee, Seung-Sup Kim

**Affiliations:** 1 Department of Environmental Health Sciences, Graduate School of Public Health, Seoul National University, Republic of Korea.; 2 Department of Biological Sciences, University of Quebec at Montreal, Canada.; 3 Department of Public Health Sciences, Graduate School of Public Health, Seoul National University, Republic of Korea.; 4 Institute of Health and Environment, Seoul National University, Republic of Korea.

**Keywords:** chemical exposure, gender disparity, occupational health, physical exposure, psychosocial exposure

## Abstract

**Objective:**

Occupational health researchers have often treated gender as a confounder in epidemiologic studies, but gender may influence exposure profiles. This study investigated gender differences in occupational hazard exposures within the same occupation.

**Methods:**

We analyzed the 6^th^ Korean Working Conditions Survey (2020), a nationally representative dataset from South Korea. After restricting the study population to 22 511 full-time wage workers, we assessed 18 self-reported occupational exposures (4 physical, 4 chemical, 1 biological, 6 musculoskeletal, 3 psychosocial). To create matched samples, each man was matched with a woman in the same occupational and industrial codes using 'nearest neighbor matching' based on the propensity scores, considering age, education, employment status, the number of subordinates, and company size. This resulted in a matched study population of 3918 male and 3918 female workers in 403 occupations. Conditional logistic regression was applied to examine gender differences within the same occupation, adjusting for other covariates.

**Results:**

We found persistent gender differences in occupational hazard exposures, even after matching of men and women within the same occupation and industry based on propensity scores. Men reported a higher prevalence of exposure to physical (eg, loud noise) and chemical factors (eg, chemical products), while women were more likely to be exposed to psychosocial factors (eg, handling angry clients). The findings on musculoskeletal factors were mixed, with men being more exposed to standing and women to repetitive hand movements.

**Conclusions:**

Gender should be considered when planning interventions to reduce occupational harmful exposures, even within the same occupation.

Gender disparities in occupational health are a crucial issue in public health. For example, research conducted in New York State has shown that the number of male deaths due to work-related diseases is more than triple that of females (1). The US had an incidence rate of 114 nonfatal occupational injuries and illnesses involving days away from work per 100 000 full-time male workers as compared to 92 per 100 000 full-time female workers in 2015 (2). A study in Australia also reported disparities between genders in workplace injury claims: Males had a 1.4 times higher rate of physical injury claims compared to females, whereas females had a 1.9 times higher rate of mental disorder claims compared to their male counterparts (3). In the Canadian province of Quebec, women report 55% more non-traumatic musculoskeletal disorders compared to men (4). These data highlight the need to acknowledge and address the diverse obstacles male and female workers encounter.

Although differences in recognizing occupational health damage between women and men may contribute to these discrepancies (5–7), gender disparities in occupational health outcomes may be primarily attributed to the unequal distribution of women and men across occupational categories (3, 8). This gender segregation of occupations may account to some extent for differential exposure to workplace hazards. According to an International Labor Organization report on job distribution in 121 countries in 2020, men were primarily employed in the construction and metal and electrical craft sectors, where workers were more likely to be exposed to physical and chemical occupational risks (9). More women were employed in the healthcare, service, and sales sectors, where psychosocial risk factors were prevalent (9). However, it has been reported that, even within the same occupation, male and female workers were exposed to different risks because they performed different tasks (10–13).

Several studies have investigated gender differences in occupational hazard exposures within the same occupational groups (13–21). A detailed analysis of work activity revealed that the tasks of males and females differ even when they have the same occupations. For instance, in Canadian hospitals, the roles of cleaners were classified as ‘light’ or ‘heavy’ tasks, often aligned with gender norms; ‘light’ work was usually performed by female cleaners, including bending and reaching, repetitive upper limb movements, and handling light weights. On the other hand, ‘heavy’ work, done mainly by male cleaners, more often involved neutral postures, upper limb movements, and pushing mops weighing 1-6 kg (14).

Although previous studies provided valuable insights into the distinct experiences of males and females within the same occupation, most were limited to specific occupations or types of exposures (13–20). However, one study that comprised a broad range of occupations in New Zealand included a comprehensive list of occupational exposures, including physical, chemical, and psychosocial risk factors. Eng et al (21) investigated gender differences across 151 occupational groups using matched samples of 604 males and 604 females who reported working in the same occupation. Males reported higher levels of exposure to physical and chemical risks such as welding fumes, herbicides, wood dust, solvents, and vibrating tools (21). In contrast, females were more likely to report musculoskeletal risks like repetitive movements, working at high speed, and awkward or tiring postures (21).

This research had several limitations. First, it did not include psychosocial occupational hazards caused by clients or patients. Furthermore, it used occupational categories alone (without respect to industry) when matching males and females. Therefore, this approach may have grouped heterogeneous occupations under a single occupational classification. For example, manufacturing plant and office building cleaning workers may be exposed to different occupational hazards. A cleaner in a manufacturing plant may be more frequently exposed to hazardous waste, whereas a cleaning worker in an office building might primarily deal with dust and musculoskeletal risks associated with repetitive tasks, with fewer exposures to hazardous materials other than the cleaning solutions.

To understand how occupational exposures differ between male and female workers within the same occupations and the same industries in South Korea, we analyzed a nationally representative dataset. We assessed 18 occupational risk factors, including physical, chemical, biological, musculoskeletal, and psychosocial hazards. We then examined gender differences in the 18 occupational exposures, after matching male and female workers within the same occupation and industry by combining administrative information on occupational and industrial codes.

## Methods

### Study population.

**Figure 1:**
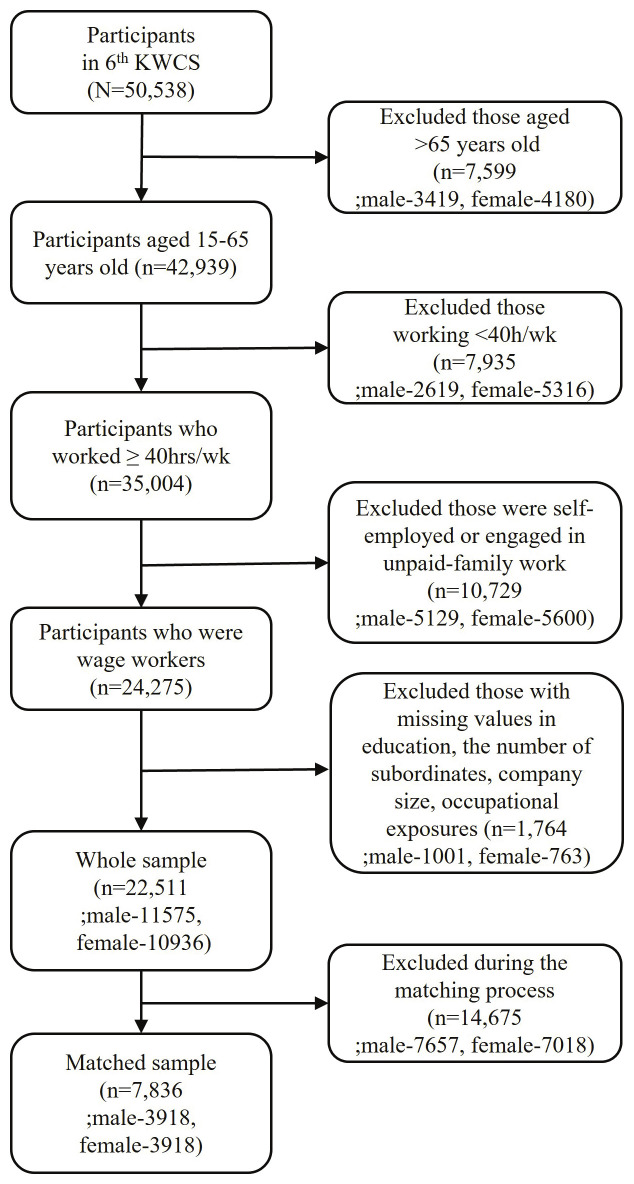
Flow diagram of study population selection from 6th KWCS.

We analyzed a nationally representative dataset of Korean workers from the 6^th^ Korean Working Conditions Survey (KWCS), administered by the Korean Occupational Safety and Health Agency in 2020. The KWCS aimed to investigate various work environments affecting occupational health and safety. A multistage random sampling method was used based on enumeration districts from the 2020 Population and Housing Census. KWCS included 50 538 economically active participants aged ≥15 years. Among these participants, we excluded those >65 years old (N=7599), aligning with the average retirement age in OECD countries (22). We focused on full-time wage workers and those working ≥40 hours per week (N=24 275), following the Korean Labor Standards Act criteria for full-time employment. We also excluded participants with missing information on covariates (N=1764). The final analysis included a total of 22 511 individuals. We utilized self-reported sex at birth as a surrogate measure for gender. To investigate gender differences within the same occupations, we applied a one-to-one matching process, resulting in a subset of 7836 participants from the overall sample (figure 1).

### Measure

Our study focused on 18 occupational hazards, including physical, chemical, biological, musculoskeletal, and psychosocial exposures. Each occupational exposure was assessed by the following questions: “Please tell me, using the following scale, are you exposed at work to …?”. The five physical hazards included (i) vibration from tools or machinery, (ii) loud noise that required the respondent to raise his/her voice to talk to people, (iii) high temperatures making him/her perspire even when not working, (iv) low temperatures whether indoors or outdoors, and (v) inhalation of substances like smoke, fumes (such as welding or exhaust fumes), powers, or dust (such as wood dust or mineral dust). The three chemical hazards were (i) breathing vapors such as solvents and thinners, (ii) handling or being in contact with chemical products or substances, and (iii) exposure to tobacco smoke from other people. One biological exposure was assessed with a question about exposure to infectious materials such as body waste, bodily fluids, and laboratory samples. Six musculoskeletal exposures were described as (i) tiring or painful positions (except standing or sitting), (ii) lifting or moving people, (iii) carrying or moving other heavy loads, (iv) standing (including walking), (v) sitting, and (vi) repetitive hand or arm movements. For three psychosocial exposures, participants were asked about their experiences of (i) dealing directly with people who are not other employees, such as customers, passengers, pupils, or patients, (ii) handling angry clients, customers, patients, or pupils, and (iii) emotionally disturbing situations. The possible responses for each of 18 items were as follows: (a) all of the time, (b) almost all of the time, (c) about three-quarters of the time, (d) about half of the time, (e) about one-quarter of the time, (f) almost never, and (g) never. The responses were categorized in a binary manner, with “exposed” indicating occurrence one quarter of the time or more (a, b, c, d, e) or “non-exposed” indicating occurrence less than one quarter of the time (f, g).

When matching occupations between male and female workers, we used industry codes (IC) and occupation codes (OC). Because occupational exposures can significantly vary within the same OC due to differences in industry settings, we used both IC and OC. IC was coded utilizing the 10^th^ Korean Standard Industrial Classification (KSIC) of 2017, and OC was coded based on the 7^th^ Korean Standard Classification of Occupations (KSCO) in 2017. We utilized two-digit IC from the KSIC, covering 77 categories, and three-digit OC from the KSCO, covering 156 categories, as provided by the KWCS. This resulted in 12 012 possible occupation combinations (calculated as 77 × 156). However, within our study population of 22 511 workers, only 1587 occupations had at least one worker in each category. After matching, the study population was reduced to 7836 workers across 403 occupations.

Sociodemographic variables included age (15–29, 30–39, 40–49, 50–59, and 60–65 years), and education (elementary school graduate or less, middle school graduate, high school graduate, and college graduate or higher). Occupational variables were employment status (permanent, temporary, or daily labor), the number of subordinates (0, 1–6, and ≥6), company size (≤49, 50–299, and ≥300 workers), and weekly working hours (40–51, 52–67, and ≥68).

### Statistical analysis

First, logistic regression was applied to investigate gender differences in occupational exposures after adjusting for covariates, including age, education, employment status, company size, and weekly working hours. We compared the prevalence of occupational exposures among female workers to that of male workers (reference group). We then investigated whether the differences in exposure between genders were solely due to differing types of occupation, or if they persisted within the same occupation. To establish matched samples, we categorized the study sample into 1587 occupations. Each male worker was matched with a female worker in the same IC and OC using the 'nearest neighbor matching' (NNM) method with a caliper of 0.2, selecting the closest unmatched subject in terms of the propensity score based on age, education, employment status, the number of subordinates, and company size (23). In order to assess the balance after matching, the standardized mean differences (SMD) were calculated (24). After the matching process, we applied conditional logistic regression with the matched sample adjusting for covariates, including age, education, employment status, company size, and weekly working hours. Results were presented as odds ratios (OR) with 95% confidence intervals (CI). For the sensitivity analysis, we conducted another NNM using the Mahalanobis distance metric instead of propensity scores. All statistical analyses were performed using R (version 4.3.0, R Foundation for Statistical Computing).

## Results

Table 1 presents the characteristics of the whole sample and the subset of males and females matched according to IC and OC. Most workers were college graduates and permanently employed. Over 60% of workers were employed by companies with 1–49 workers. About 10% of employees worked >52 hours per week. The SMD of matching covariates after matching are also shown in table 1. The residual imbalances in the variables used for matching were acceptable, since SMD <0.25 indicates a negligible variation in the distribution of covariates after matching (25).

**Table 1:** Distribution of the whole sample and matched samples.

		Whole sample					Matched sample ^†^			
		Male		Female	Standardized mean difference ^‡^		Male		Female	Standardized mean difference ^‡^
		N (%)		N (%)			N (%)		N (%)	
Age (years)					0.071					0.1947
	15–29	1540 (13.3)		1435 (13.1)			703 (17.9)		525 (13.4)	
	30–39	3207 (27.7)		2670 (24.4)			1191 (30.4)		1022 (26.1)	
	40–49	3333 (28.8)		2963 (27.1)			1041 (26.6)		1098 (28.0)	
	50–59	2582 (22.3)		3062 (28.0)			753 (19.2)		1012 (25.8)	
	60–65	913 (7.9)		806 (7.4)			230 (5.9)		261 (6.7)	
Education					0.142					0.1970
	≤Elementary school graduate	60 (0.5)		90 (0.8)			14 (0.4)		28 (0.7)	
	Middle school graduate	291 (2.5)		352 (3.2)			59 (1.5)		111 (2.8)	
	High school graduate	3601 (31.1)		3716 (34.0)			970 (24.8)		1251 (31.9)	
	≥College graduate	7623 (65.9)		6778 (62.0)			2875 (73.4)		2528 (64.5)	
Employment status					0.0763					0.0527
	Permanent	10440 (90.2)		9913 (90.6)			3638 (92.9)		3570 (91.1)	
	Temporary	720 (6.2)		906 (8.3)			245 (6.3)		311 (7.9)	
	Daily labor	415 (3.6)		117 (1.1)			35 (0.9)		37 (0.9)	
Number of subordinates					0.0234					0.0176
	0	4672 (40.4)		5156 (47.1)			1613 (41.2)		1851 (47.2)	
	1–5	6093 (52.6)		5364 (49.0)			2076 (53.0)		1877 (47.9)	
	≥6	810 (7.0)		416 (3.8)			229 (5.8)		190 (4.8)	
Company size (person)					0.3737					0.0498
	1–49	7262 (62.7)		8250 (75.4)			2772 (70.8)		2855 (72.9)	
	50–299	2600 (22.5)		2009 (18.4)			818 (20.9)		738 (18.8)	
	300–	1713 (14.8)		677 (6.2)			328 (8.4)		325 (8.3)	
Weekly working time (hours)										
	40–51	9991 (86.3)		10035 (91.8)			2810 (86.7)		3011 (92.9)	
	52–67	1398 (12.1)		816 (7.5)			387 (11.9)		208 (6.4)	
	≥68	186 (1.6)		85 (0.8)			45 (1.4)		23 (0.7)	
Occupation										
	Managers	134 (1.2)		27 (0.2)			6 (0.2)		6 (0.2)	0.000
	Professionals and related workers	2549 (22.0)		2865 (26.2)			710 (21.9)		710 (21.9)	0.000
	Clerks	2877 (24.9)		3456 (31.6)			1282 (39.5)		1282 (39.5)	0.000
	Service workers	582 (5.0)		1511 (13.8)			278 (8.6)		278 (8.6)	0.000
	Sales workers	984 (8.5)		1525 (13.9)			497 (15.3)		497 (15.3)	0.000
	Skilled agricultural, forestry and fishery workers	64 (0.6)		15 (0.1)			2 (0.1)		2 (0.1)	0.000
	Craft and related trades workers	1605 (13.9)		270 (2.5)			60 (1.9)		60 (1.9)	0.000
	Equipment, machine operating and assembling workers	1869 (16.1)		510 (4.7)			271 (8.4)		271 (8.4)	0.000
	Elementary workers	911 (7.9)		757 (6.9)			136 (4.2)		136 (4.2)	0.000

^†^ Each male was matched one-to-one with a female in the same industry codes (IC) and occupation codes (OC) using Nearest Neighbor Matching based on age, education, employment status, the number of subordinates, and company size. ^‡^ The standardized mean differences were described to measure the level of imbalance between male and female groups.

Table 2 presents the distribution of 18 occupational exposures among both genders over the whole sample (N=22 511) and the matched sample (N=7836). In the total sample, male workers had a significantly higher exposure to physical, chemical, and biological hazards than their female counterparts (OR 1.7–4.7). In contrast, female workers were more likely to experience musculoskeletal exposures such as lifting or moving people (OR 0.56, 95% CI 0.05–0.62) and repetitive hand movements (OR 0.93, 95% CI 0.88–0.98). In addition, female workers showed a higher probability of encountering psychosocial stressors, including dealing directly with people, handling angry clients, and emotionally disturbing situations.

**Table 2:** Exposure prevalence of males and females. [OR=odds ratio; CI=confidence interval.]

Occupational hazards		Whole sample					Matched sample ^†^			
		Male (%)	Female (%)	OR	95% CI		Male (%)	Female (%)	OR	95% CI
Physical										
	Vibration	30.5	11.1	3.87	3.58−4.18		16.3	12.7	1.50	1.28−1.77
	Loud noise	21.3	8.5	3.05	2.80−3.33		12.1	8.9	1.52	1.27−1.81
	High temperature	15.7	6.4	2.90	2.63−3.20		9.4	6.5	1.74	1.41− 2.16
	Low temperature	14.7	5.1	3.46	3.11−3.84		7.7	5.4	1.70	1.36−2.13
	Smoke, fumes, powder or dust	19.6	5.4	4.68	4.23−5.17		9.6	6.2	1.83	1.48− 2.25
Chemical										
	Vapors	7.4	2.0	3.99	3.41−4.66		3.9	2.3	1.96	1.42−2.69
	Chemical products	7.3	3.2	2.47	2.16−2.82		4.0	3.1	1.40	1.06−1.85
	Tobacco smoke	7.1	2.1	3.50	3.00−4.09		3.6	2.4	1.83	1.31−2.54
Biological										
	Infection	3.8	2.3	1.72	1.47−2.03		2.2	1.6	1.56	2.29−1.06
Ergonomic										
	Tiring or painful position	34.9	32.3	1.12	1.06−1.19		30.5	29.4	1.05	0.94−1.17
	Lifting or moving people	5.5	9.8	0.56	0.50−0.62		5.6	5.2	1.07	0.84−1.36
	Heavy loads	32.2	21.0	1.96	1.83− 2.09		26.8	20.1	1.80	1.56− 2.07
	Standing	60.4	59.6	1.07	1.01−1.13		60.3	54.3	1.49	1.32−1.69
	Sitting	77.4	79.0	0.89	0.83−0.95		77.8	80.3	0.75	0.65−0.86
	Repetitive hand movements	59.6	61.6	0.93	0.88−0.98		59.7	61.8	0.90	0.81−0.99
Psychosocial										
	Dealing directly with people	41.1	55.9	0.54	0.51−0.57		50.9	50.9	0.96	0.86−1.07
	Handling angry clients	13.1	19.8	0.60	0.56−0.65		16.0	17.4	0.85	0.75−0.98
	Emotionally disturbing situation	9.4	12.1	0.72	0.66−0.79		9.4	10.5	0.82	0.70−0.96

^†^ Each male was matched one-to-one with a female in the same industry codes (IC) and occupation codes (OC) using Nearest Neighbor Matching based on age, education, employment status, the number of subordinates, and company size.

Males and females in the same occupation were matched considering age, education, employment status, the number of subordinates, and company size. Consequently, 14 675 participants were excluded from the matched analyses. We observed that matching on IC and OC attenuated gender disparities in occupational exposures. Nonetheless, the results from the matched-sample analysis showed trends similar to those observed in the whole sample, and the gender differences in most exposures remained statistically significant. The sensitivity analysis, conducted using an alternative matching method, was consistent with the main findings (supplementary material, https://www.sjweh.fi/article/4204, table S1) For most physical, chemical, and biological hazards, male workers consistently showed a higher prevalence than female workers, even after the matching process. For instance, within the whole sample, male workers were more likely to be exposed to chemical agents like vapors than female workers (OR 3.99, 95% CI 3.41–4.66). This disparity was attenuated in the matched sample but remained statistically significant (OR 1.96, 95% CI 1.42–2.69).

On the other hand, for some occupational exposures, such as standing posture and repetitive hand movements, gender differences widened after the matching process. However, with exposure to lifting or moving people, it was observed that the direction of gender differences was reversed following the matching procedure, although the gender difference within the same occupational group was not statistically significant. Over the whole sample, female workers were initially observed to be more likely than males to report exposure to lifting people (OR 0.56, 95% CI 0.50–0.62). However, after matching occupations, male workers showed a tendency for a higher prevalence of exposure to lifting or moving people than females, although the gender difference was not statistically significant (OR 1.07, 95% CI 0.84–1.36).

## Discussion

This study found significant gender disparities in 18 occupational exposures after matching occupations and industries and controlling for variables such as age, education, employment status, the number of subordinates, company size and weekly working hours. Male workers had higher odds of being exposed to physical, chemical, and biological hazards (OR 1.4–2.0) compared to their female counterparts. On the other hand, female workers were more likely to encounter some musculoskeletal and psychosocial hazards.

Our findings on gender differences can be explained in several ways. First, task allocation within the same occupation could differ between males and females, resulting in different occupational exposures for each gender (14, 15). For example, Dumais et al (26) found that in a bakery processing line, female workers manually wrapped cookies and placed them in small boxes, while males operated large mixing machines and ovens, packed the small boxes into larger ones, and loaded them onto trucks. Similarly, in factory settings, female manufacturing workers typically engaged in low-force and high-repetitive work like product packaging at rates exceeding 20 times per minute. Meanwhile, male manufacturing workers were more likely to engage in high-force and low-repetitive work, such as loading packages, at most 2 times per minute (15).

Furthermore, even when sharing the same job title and doing identical activities, male and female workers may experience varying occupational exposure levels due to distinct physical and physiological responses to the work environment. Wendela et al (17) analyzed video-recorded work processes of employees in several job categories such as care work, laboratory work, cooking, and administration. They observed that males displayed a higher frequency of neck flexions than females, while females elevated their arms more frequently than males. These differences could be attributed to the sex difference in average height. Laperriere et al (16) also reported that male and female food servers in the same restaurant displayed different walking habits. The pace of female servers was 83% faster than that of males, and female servers walked three times farther per workday than male servers. The physical difference in leg length (walking speed) and arm length (capacity to carry plates stacked on one’s arms) between males and females could partly explain these results (27).

It is important to consider who was excluded when restricting the study population to address our research question. The following two factors illustrate the severity of gender segregation across occupations. First, to focus on full-time wage workers, we excluded individuals working <40 hours per week, resulting in the removal of 2 619 males and 5 319 females (figure 1). In this process, the number of excluded females was more than twice that of males. Furthermore, while there were 1 587 occupations in the whole sample, only 403 occupations remained after NNM to examine gender differences within the same occupation. A total of 1 184 occupations were removed because they lacked sufficient numbers of opposite-gender coun terparts in male- or female-dominated occupations. The excluded major male- and female-dominated occupations are presented in supplementary tables S2 and S3.

### Limitations

First, some relevant occupational hazards might be overlooked, although we comprehensively examined various exposures, including physical, chemical, biological, musculoskeletal, and psychosocial factors that were covered in the questionnaire of the KWCS. Using a predefined list of exposures might miss some less obvious but still important risks, particularly those that are emergent or less recognized in traditional occupational health frameworks. For example, factors such as sexual harassment at work (28) or work schedules interfering with work-family life (29), which are known to differ significantly between males and females and could influence occupational health outcomes, might have needed to be included.

Secondly, the assessment of several occupational exposures in this study could have been more rigorous. For instance, the European Agency for Safety and Health at Work identified prolonged standing as an occupational hazard that can lead to negative health outcomes, such as lower back and leg pain, fatigue, and complications during pregnancy (30, 31). In their reports, prolonged standing was characterized by either standing for more than one hour continuously or accumulating more than four hours per day (30). However, this study only measured the total standing time without considering whether it involved continuous standing. As a result, a worker who stood for one continuous hour a day could have been classified as unexposed to standing, simply because their total standing time was less than one-fourth of the total working hours.

Thirdly, we measured only the length of time individuals were exposed to occupational hazards rather than the intensity of the exposure. This approach has the potential to hide variations among workers giving the same answers. For example, previous research found that a male worker in a poultry processing factory “exposed to low temperatures” might usually work at a temperature of 20–22 degrees Celsius but frequently enter and exit a freezer where the temperature was -20 degrees, while a female worker “exposed to low temperatures” in the same factory might be constantly exposed to a work environment with a usual temperature of -4 degrees Celsius (32). In this case, our measurement allows us to determine the duration of exposure to low temperatures, but it does not allow us to quantify the precise extent of the low temperature. Therefore, the male and female workers may encounter distinct low-temperature conditions, even if both workers reported experiencing “low temperatures.”

Furthermore, we analyzed only self-reported occupational exposures rather than data obtained from direct monitoring or administrative records. If males and females systematically report exposures differently, this could contribute to the observed gender differences in our study (33). For example, Hansson et al (34) found that men had higher time-weighted arm elevation exposures than women in the same occupation based on worktask diaries, while men reported lower arm elevation exposure levels than women in surveys. These limitations emphasize the need for a broader and more detailed occupation risk assessment in future research to better understand occupational exposure differences between males and females.

Lastly, despite considering the 12 012 possible occupation categories and analyzing 1587 occupations in the whole sample and 403 occupations in the matched sample, heterogeneity may still remain within occupation categories. To more accurately examine gender differences in occupational exposures within the same occupation, future research should utilize more detailed occupational categories to enhance matching precision.

### Concluding remarks

This study analyzed nationally representative data from South Korean workers. We found that gender differences in occupational exposures occur overall in the full-time working population and persist even within the same occupation and employment sector. Our findings imply that it is important to consider gender in the design and implementation of occupational health policies and practices. Gender-sensitive intervention strategies to make safer and healthier work environments would be more effective and beneficial for both male and female workers.

### Statement of ethics approval

This research study was conducted retrospectively using publicly data with permission from KOSHA. Informed consent was not required to use the dataset. This study was exempted from Institutional Review Board approval by Seoul National University (IRB No. E2310/002-001).

## Supplementary material

Supplementary material

## References

[1] Lax MB, Zoeckler JM. Occupational disease in New York State: an update. New Solut 2023 Feb;32(4):304–23. 10.1177/10482911231152896 36799954

[2] Bureau Of Labor Statistics. Nonfatal occupational injuries and illnesses requiring days away from work, 2015. US Department of Labor. 2016.

[3] Berecki-Gisolf J, Smith PM, Collie A, McClure RJ. Gender differences in occupational injury incidence. Am J Ind Med 2015 Mar;58(3):299–307. 10.1002/ajim.22414 25641425

[4] A million Quebecers suffer from work-related musculoskeletal disorders. The Canadian Press. 2020 Jan. Available from: https://montreal.ctvnews.ca/a-million-quebecers-suffer-from-work-related-musculoskeletal-disorders-1.4756251

[5] Lippel K. Compensation for musculoskeletal disorders in Quebec: systemic discrimination against women workers? Int J Health Serv 2003;33(2):253–81. 10.2190/JPQD-RT1G-QKTK-JF2R 12800887

[6] Lippel K. Workers’ compensation and stress. Gender and access to compensation. Int J Law Psychiatry 1999;22(1):79–89. 10.1016/S0160-2527(98)00019-3 10086293

[7] Leijon O, Lindahl E, Torén K, Vingård E, Josephson M. First-time decisions regarding work injury annuity due to occupational disease: a gender perspective. Occup Environ Med 2014 Feb;71(2):147–53. 10.1136/oemed-2013-101489 24142986

[8] McDiarmid M, Oliver M, Ruser J, Gucer P. Male and female rate differences in carpal tunnel syndrome injuries: personal attributes or job tasks? Environ Res 2000 May;83(1):23–32. 10.1006/enrs.2000.4042 10845778

[9] These occupations are dominated by women 2020 [Internet]. United Nations: International Labour Organization department of statistics; c1996-2024 [cited 2024 Sep 12]. Available from: https://ilostat.ilo.org/blog/these-occupations-are-dominated-by-women/

[10] Messing K, Dumais L, Courville J, Seifert AM, Boucher M. Evaluation of exposure data from men and women with the same job title. J Occup Med 1994 Aug;36(8):913–7. 7807275

[11] Wuytack F, Evanoff B, Dale AM, Gilbert F, Fadel M, Leclerc A et al. Comparing physical work exposures between men and women: findings from 65 281 workers in France. Occup Environ Med 2023 Oct;80(10):558–63. 10.1136/oemed-2023-108839 37770181

[12] Wuytack F, Evanoff BA, Dale AM, Gilbert F, Fadel M, Leclerc A et al. Comparison between musculoskeletal pain and gender-specific, non-gendered job-exposure matrix and self-reported exposures in CONSTANCES. J Occup Rehabil 2024 Sep;34(3):594–605. 10.1007/s10926-023-10148-w 37932500

[13] Biswas A, Harbin S, Irvin E, Johnston H, Begum M, Tiong M et al. Sex and gender differences in occupational hazard exposures: a scoping review of the recent literature. Curr Environ Health Rep 2021 Dec;8(4):267–80. 10.1007/s40572-021-00330-8 34839446 PMC8627292

[14] Messing K, Chatigny C, Courville J. ‘Light’ and ‘heavy’ work in the housekeeping service of a hospital. Appl Ergon 1998 Dec;29(6):451–9. 10.1016/S0003-6870(98)00013-1 9796791

[15] Silverstein BA, Fine LJ, Armstrong TJ. Hand wrist cumulative trauma disorders in industry. Br J Ind Med 1986 Nov;43(11):779–84. 10.1201/b12565-3 3790459 PMC1007752

[16] Laperrière E, Ngomo S, Thibault MC, Messing K. Indicators for choosing an optimal mix of major working postures. Appl Ergon 2006 May;37(3):349–57. 10.1016/j.apergo.2005.06.014 16182229

[17] Hooftman WE, van der Beek AJ, van de Wal BG, Knol DL, Bongers PM, Burdof A et al. Equal task, equal exposure? Are men and women with the same tasks equally exposed to awkward working postures? Ergonomics 2009 Sep;52(9):1079–86. 10.1080/00140130902915921 19606366

[18] Carey RN, Glass DC, Peters S, Reid A, Benke G, Driscoll TR et al. Occupational exposure to solar radiation in Australia: who is exposed and what protection do they use? Aust N Z J Public Health 2014 Feb;38(1):54–9. 10.1111/1753-6405.12174 24494947

[19] Newman CJ, de Vries DH, d’Arc Kanakuze J, Ngendahimana G. Workplace violence and gender discrimination in Rwanda’s health workforce: increasing safety and gender equality. Hum Resour Health 2011 Jul;9:19. 10.1186/1478-4491-9-19 21767411 PMC3154143

[20] Guytingco A, Thepaksorn P, Neitzel RL. Prevalence of abnormal serum cholinesterase and associated symptoms from pesticide exposure among agricultural workers in the south of Thailand. J Agromedicine 2018;23(3):270–8. 10.1080/1059924X.2018.1470049 30047860

[21] Eng A, ’t Mannetje A, McLean D, Ellison-Loschmann L, Cheng S, Pearce N. Gender differences in occupational exposure patterns. Occup Environ Med 2011 Dec;68(12):888–94. 10.1136/oem.2010.064097 21486991

[22] OECD. Pensions at a Glance 2021: OECD and G20 Indicators: Organisation for Economic Co-operation and Development OECD; 2021.

[23] Austin PC. A comparison of 12 algorithms for matching on the propensity score. Stat Med 2014 Mar;33(6):1057–69. 10.1002/sim.6004 24123228 PMC4285163

[24] Zhang Z, Kim HJ, Lonjon G, Zhu Y; written on behalf of AME Big-Data Clinical Trial Collaborative Group. Balance diagnostics after propensity score matching. Ann Transl Med 2019 Jan;7(1):16. 10.21037/atm.2018.12.10 30788363 PMC6351359

[25] Stuart EA. Matching methods for causal inference: A review and a look forward. Stat Sci 2010 Feb;25(1):1–21. 10.1214/09-STS313 20871802 PMC2943670

[26] Dumais L, Messing K, Seifert AM, Courville J, Vezina N. Make me a cake as fast as you can: forces for and against change in the sexual division of labour at an industrial bakery. Work Employ Soc 1993;7(3):363–82. 10.1177/095001709373002

[27] Gillespie RM, Herbert R, Punnett L. Chapter 41—work-related musculo-skeletal disorders. Women and Health (Second Edition) edited by Marlene B Goldman, Rebecca Troisi and Kathryn M Rexrode, Academic Press. 2013:613-28.

[28] Kim HR. Associations between workplace violence, mental health, and physical health among Korean Workers: The Fifth Korean Working Conditions Survey. Workplace Health Saf 2022 Mar;70(3):161–72. 10.1177/21650799211023863 34323126

[29] Choi SM, Kim CW, Park HO, Park YT. Association between unpredictable work schedule and work-family conflict in Korea. Ann Occup Environ Med 2023 Nov;35:e46. 10.35371/aoem.2023.35.e46 38148922 PMC10751215

[30] Peereboom K. Langen Nd, Bortkiewicz A. Prolonged constrained standing at work. European Agency for Safety and Health at Work; 2021.

[31] Waters TR, Dick RB. Evidence of health risks associated with prolonged standing at work and intervention effectiveness. Rehabil Nurs 2015;40(3):148–65. 10.1002/rnj.166 25041875 PMC4591921

[32] Messing K. Bent Out of Shape: Shame, Solidarity, and Women’s Bodies at Work: Between the Lines; 2021. Chapter 12.

[33] Hooftman WE, van der Beek AJ, Bongers PM, van Mechelen W. Gender differences in self-reported physical and psychosocial exposures in jobs with both female and male workers. J Occup Environ Med 2005 Mar;47(3):244–52. 10.1097/01.jom.0000150387.14885.6b 15761320

[34] Hansson GA, Balogh I, Byström JU, Ohlsson K, Nordander C, Asterland P et al.; Malmö Shoulder-Neck Study Group. Questionnaire versus direct technical measurements in assessing postures and movements of the head, upper back, arms and hands. Scand J Work Environ Health 2001 Feb;27(1):30–40. 10.5271/sjweh.584 11266144

