# Supplementary material for: Gender differences in occupational hazard exposures within the same occupation: A nationally representative analysis in South Korea
Source: Scand J Work Environ Health. 2025 Feb 28;51(2):111–8. doi: 10.5271/sjweh.4204 (PMC12947313; doi:10.5271/sjweh.4204)
Supplement: Supplementary material [file SJWEH-51-111-S001.pdf]

Gender differences in occupational hazard exposures within the same occupation: A nationally representative analysis in South Korea[1]

by Garin Lee, MPH, Karen Messing, PhD, Woojoo Lee, PhD, Ji-Hwan Kim, PhD, Hayoung Lee, MPH, Seung-Sup Kim, ScD[2]

1. Supplementary material
2. Correspondence to: Seung-Sup Kim, MD, MPH, ScD, Department of Environmental Health Sciences, Graduate School of Public Health, Seoul National University, 1 Gwanak-ro, Daehak-dong, Gwanak-gu, Seoul 08826, Republic of Korea. [E-mail: kim.seungsup@snu.ac.kr]

Supplementary Table 1. Exposure prevalence of males and females in matched sample with robust-Mahalanobis distance

Occupational Hazards	Matched sample			
	Male (%)	Female (%)	OR	95% CI
Physical				
Vibration	15.3	12.2	1.45	1.22–1.73
Loud noise	12.0	9.7	1.30	1.08–1.55
High temperature	8.3	5.6	1.68	1.33–2.12
Low temperature	7.1	4.8	1.67	1.31–2.13
Smoke, fumes, powder or dust	8.8	6.2	1.63	1.29–2.06
Chemical				
Vapors	3.5	2.0	1.97	1.36–2.86
Chemical products	3.9	2.7	1.68	1.21–2.32
Tobacco smoke	3.4	2.2	1.74	1.24–2.43
Biological				
Infection	2.3	1.6	1.73	1.09–2.76
Ergonomic				
Tiring or painful position	30.3	30.8	0.96	0.85–1.07
Lifting or moving people	5.9	5.3	1.16	0.91–1.47
Heavy loads	25.7	18.8	1.65	1.43–1.90
Standing	59.6	53.5	1.52	1.32–1.74
Sitting	78.6	80.4	0.89	0.77–1.02
Repetitive hand movements	58.2	60.6	0.90	0.81–1.00
Psycholosocial				
Dealing directly with people	146.6	188.5	0.99	0.88–1.11
Handling angry clients	16.9	19.2	0.84	0.73–0.97
Emotionally disturbing situation	10.1	11.8	0.83	0.71–0.98

Supplementary Table 2. Male-dominated occupations omitted in the matched population due to absence of female counterparts

**Unmatched worker (n)**		Korean Standard Classification of Occupations (code number)	Korean Standard Industrial Classification (code number)
**Male**	**Female**		
67	0	Automobile Drivers (873)	Wholesale trade on own account or on a fee or contract basis (46)
60	0	Plumbers (792)	Specialized construction activities (42)
53	0	Construction Related Technical Workers (782)	General construction (41)
47	0	Machinery Equipment Fitters and Mechanics (753)	Specialized construction activities (42)
43	0	Electrical and Electronic Equipment Operators (862)	Real estate activities (68)
36	0	Broadcasting and Telecommunications Equipment Related Fitters and Repairers (772)	Specialized construction activities (42)
27	0	Machinery Equipment Fitters and Mechanics (753)	Manufacture of electronic components, computer; visual, sounding and communication equipment (26)
26	0	Power Generation and Distribution Equipment Operators (861)	Electricity, gas, steam and air conditioning supply (35)
25	0	Machinery Equipment Fitters and Mechanics (753)	Maintenance and repair services of industrial machinery and equipment (34)
24	1*	Machinery Equipment Fitters and Mechanics (753)	Manufacture of other machinery and equipment (29)
22	0	Construction Related Technical Workers (782)	Business support services (75)

* No suitable female match was found within the specified caliper of 0.2, because matching was based on propensity scores, calculated from age, education, employment status, number of subordinates, and company size.

Supplementary Table 3. Female-dominated occupations omitted in the matched population due to absence of male counterparts

Unmatched worker (n)		Korean Standard Classification of Occupations (code number)	Korean Standard Industrial Classification (code number)
**Male**	**Female**		
0	27	Domestic Chores and Infant Rearing Helpers (951)	Activities of households as employers of domestic personnel (97)
1*	22	Designers (285)	Manufacture of wearing apparel, clothing accessories and fur articles (14)
1*	20	Accounting and Book-keeping Clerks (313)	Architectural, engineering and other scientific technical services (72)
0	15	Store Sales Workers (521)	Warehousing and support activities for transportation (52)
0	14	Dietitians (244)	Human health activities (86)
0	14	Caregiving and Health Service Workers (421)	Other personal services activities (96)
0	12	Dietitians (244)	Education (85)
0	12	Customer Service and Other Office Clerks (399)	Real estate activities (68)
1*	11	Cleaners and Sanitation Workers (941)	Accommodation (55)
0	10	Nurses (243)	Education (85)

* No suitable male match was found within the specified caliper of 0.2, because matching was based on propensity scores, calculated from age, education, employment status, number of subordinates, and company size.
